# MicroRNA-25-5p negatively regulates TXNIP expression and relieves inflammatory responses of brain induced by lipopolysaccharide

**DOI:** 10.1038/s41598-022-21169-5

**Published:** 2022-10-26

**Authors:** Jiabing Wang, Zhinan Ye, Yuan Chen, Xinyu Qiao, Yong Jin

**Affiliations:** 1 Department of Pharmacy, Municipal Hospital Affiliated to Taizhou University, Taizhou, 318000 China; 2 Department of Neurology, Municipal Hospital Affiliated to Taizhou University, Taizhou, 318000 China; 3 Department of Neurosurgery, Municipal Hospital Affiliated to Taizhou University, Taizhou, 318000 China

**Keywords:** Immunology, Molecular biology

## Abstract

Sepsis is one of the most common causes of death in patients suffering from severe infection or injury. Currently, a specific effective therapy remains to be established. In the present study, miR-25-5p, miR-105, miR-106b-5p, miR-154-3p, miR-20b-5p, miR-295-3p, miR-291-3p, miR-301b, miR-352, and miR-93-5p were predicted to target TXNIP mRNA from the databases of miRDB, Targetscan, and microT-CDS. The luciferase reporter assay confirmed that miR-25-5p negatively regulates *TXNIP* expression. The ELISA analyses and western blotting demonstrated that miR-25-5p downregulated the production of IL-1β, IL-6, IL-8, and TNF-α in lipopolysaccharide (LPS)-stimulated cells or rats, as well as the protein levels of *TXNIP*, NLRP3, and cleaved caspase-1. In addition, miR-25-5p increased the cell viability and decreased the apoptosis in LPS-stimulated CTX TNA2 cells and reduced the abnormal morphology of the brain in LPS-stimulated rats. Besides, miR-25-5p decreased the relative mean fluorescence intensity of DCF in LPS-stimulated CTX TNA2 cell, apoptosis, and protein levels of *MnSOD* and catalase in LPS-stimulated brains. These findings indicate that miR-25-5p downregulated LPS-induced inflammatory responses, reactive oxygen species production, and brain damage, suggesting that miR-25-5p is a candidate treatment for septic encephalopathy.

## Introduction

Sepsis commonly occurs in patients suffering from severe infection and injury. Sepsis syndrome is characterized by an excessive inflammatory response to infection and the inflammatory response inhibiting homeostasis^1^. The dysregulated inflammatory response is thought to be a result of heterogeneous microbial pathogens, specifically for gram-negative bacteria^2^. In severely affected patients, the dysregulated inflammatory response leads to cardiovascular, renal, and hepatic dysfunction, which are known as sepsis shock, which typically have high morbidity and mortality^3,4^. The excessive systemic inflammatory response can further cause inflammatory reactions in the central nervous system and brain injury, such as sepsis-associated encephalopathy or septic encephalopathy^5^. Unfortunately, a specific effective therapy currently remains to be established, although efforts have been made for decades. Nonetheless, previous findings on the pathological mechanism of sepsis indicate that the dysregulated inflammatory response of the host immune system is the critical pathological process involved in the development of sepsis^6^. A specific therapy targeting the pathological pathway of an excessively sustained inflammatory response may be an effective candidate.

MicroRNA (miRNA) is a non-coding single-stranded RNA that is composed of 18–25 nucleotides. The miRNA has been proven to be dedicated to expression regulation in the post-transcription level in organisms^7^. Typically, the miRNA binds to the 3’-UTR sequence of mRNA completely or partially, leading to the downregulation of the expression level of target mRNA^8^. Currently, many miRNAs have had their regulative activities in inflammation and immunity confirmed^9,10^. For example, miR-10a targets multiple key regulative components in the NF-κB signaling pathway, and the target genes include TAK 1, β-TrCP, and MAP3K7^11,12^. miR-21 targets programmed cell death 4 and negatively regulates the TLR4 signaling pathway^13,14^. miR-24 can decrease the production of TNF-α and IL-6 by the suppression of HMGB1/NF-κB-associated inflammatory signaling^15^. miR-124 downregulates TLR6, MyD88, TNF-α, and TRAF6 of TLR signaling^16,17^. miR-146 and miR-155 play center regulative roles in inflammation and immunity^18,19^.

The thioredoxin-interacting protein (TXNIP)/pyrin domain-containing 3 (NLRP3) pathway has been proven to be associated with the development of septic encephalopathy in recent years^20^. The NLRP3 inflammasome activates the production of caspase-1 and stimulates the secretion of IL-1β and IL-18^21,22^. The goal of the present study was to investigate a candidate therapy of miRNA for septic encephalopathy. We first screened miRNAs that potentially target *TXNIP* mRNA from miRNA databases and verify their regulative function in *TXNIP* expression. Secondly, we explore the function of the miRNA mimic in LPS-stimulated CTX TNA2 cells. Finally, the therapeutic function of the miRNA was further verified using in vivo experiments.

## Results

### miR-25-5p negative regulates *TXNIP* expression

**Figure 1:**
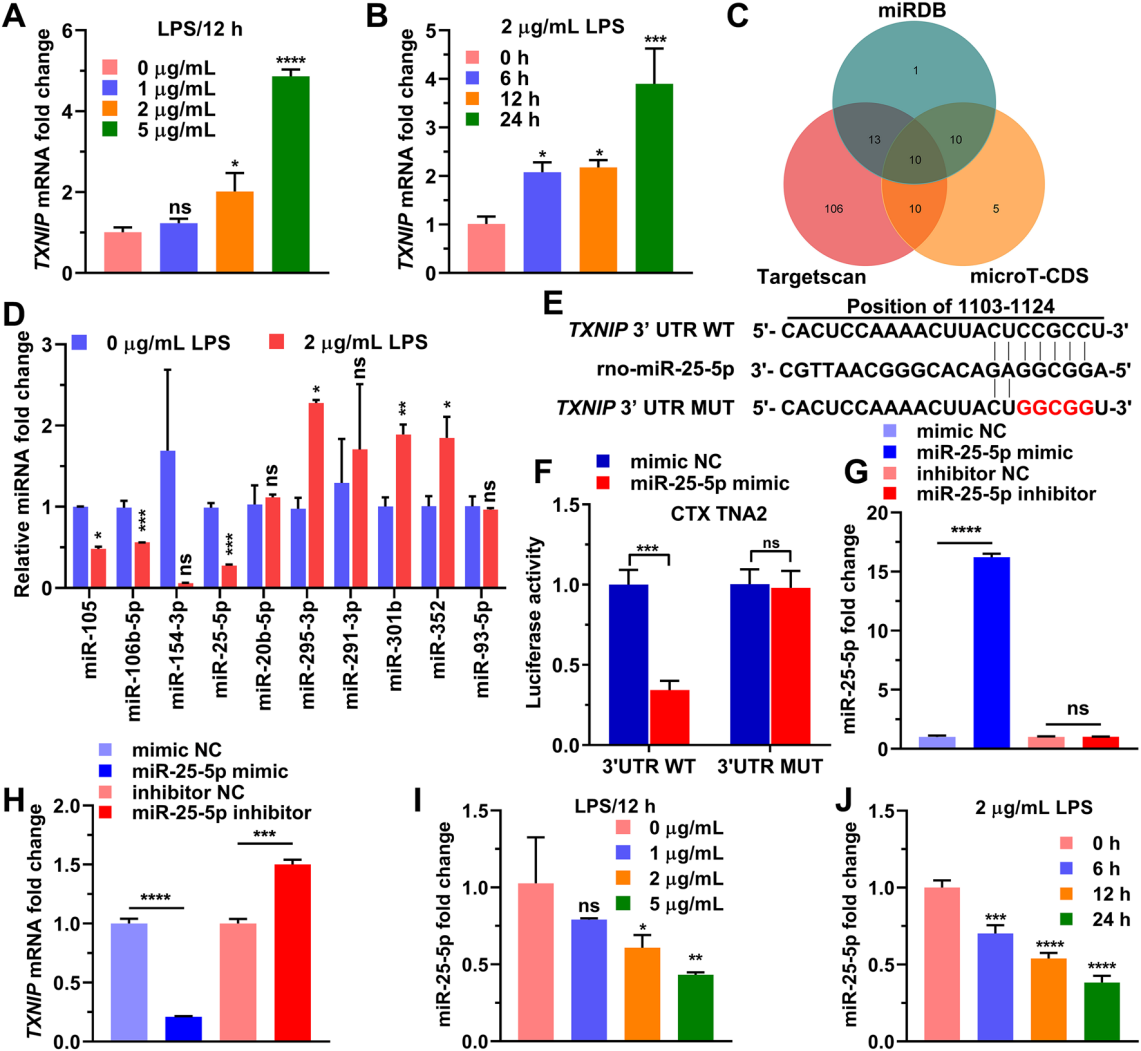
miR-25-5p negatively regulates *TXNIP* expression. (**A**) *TXNIP* fold change of CTX TNA2 cells in response to 0 μg/mL, 1 μg/mL, 2 μg/mL, and 5 μg/mL LPS stimulation over 12 h. (**B**) *TXNIP* fold change of CTX TNA2 cells in response to 2 μg/mL LPS stimulation over 0 h, 6 h, 12 h, and 24 h. (**C**) Numbers of miRNAs predicted to bind to 3′-UTR sequence of *TXNIP* mRNA from miRDB, microT-CDS, and Targetscan. (**D**) The fold changes of top 10 predicted miRNAs CTX TNA2 cells in response to 2 μg/mL LPS stimulation. (**E**) Sequences of wild-type (*TXNIP* 3′UTR WT) and mutant *TXNIP* 3′-UTR (*TXNIP* 3′UTR MUT). (**F**) Luciferase activities of recombined reporter plasmids (*TXNIP* 3′UTR WT and *TXNIP* 3′UTR MUT) co-transfected with miR-25-5p mimic. (**G**, **H**) miR-25-5p (**G**) and *TXNIP* (**H**) mRNA fold change of CTX TNA2 cells in response to the transfection of miR-25-5p mimic and miR-25-5p inhibitor. (**I**) miR-25-5p fold change of CTX TNA2 cells in response to 0 μg/mL, 1 μg/mL, 2 μg/mL, and 5 μg/mL LPS stimulation over 12 h. (**J**) miR-25-5p fold change of CTX TNA2 cells in response to 2 μg/mL LPS stimulation over 0 h, 6 h, 12 h, and 24 h. The relative expression levels of miRNA and mRNA were determined by qPCR and protein levels were determined by western blotting. The independent *t*-test was applied to the comparison between groups (**D**, **F**, **G**, and **H**). One-way analysis of variance (ANOVA), followed by Tukey’s test was used to identify the significant differences among multiple groups (**A**, **B**, **I**, and **J**). Values are shown as the mean ± standard deviation (SD). Con: negative control of LPS, LPS: lipopolysaccharide, mimic NC: negative control of miR-25-5p mimic, inhibitor NC: negative control of miR-25-5p inhibitor. ns indicates not significant. *P < 0.05. **P < 0.01. ***P < 0.001. ****P < 0.0001.

We stimulated the CTX TNA2 cells with increasing lipopolysaccharide (LPS) concentration, at 1 μg/mL, 2 μg/mL, and 5 μg/mL for 12 h, and found that *TXNIP* RNA level was significantly increased with LPS at 2 and 5 μg/mL (Fig. 1A). We then stimulated cells LPS at 2 μg/mL in time course experiments from 0 to 24 h, and found that *TXNIP* RNA level was significantly at 6, 12, and 24 h (Fig. 1B). After predicting miRNAs targeting *TXNIP* from miRDB, Targetscan and microT-CDS, we found that 10 miRNAs, miR-105, miR-106b-5p, miR-154-3p, miR-25-5p, miR-20b-5p, miR-295-3p, miR-291-3p, miR-301b, miR-352, and miR-93-5p, were included in miRDB, Targetscan and microT-CDS, and all of them had the potential to target *TXNIP* (Fig. 1C). After cells were treated with 2 μg/mL LPS for 12 h, we detected the RNA level of miR-105, miR-106b-5p, miR-154-3p, miR-25-5p, miR-20b-5p, miR-295-3p, miR-291-3p, miR-301b, miR-352, and miR-93-5p, and a significant decrease in expression level was observed in miR-25-5p (*t* = 15.40, *P* < 0.0001), miR-105 (*t* = 14.55, *P* < 0.001), and miR-106b-5p (*t* = 6.68, *P* < 0.01) (Fig. 1D). We constructed plasmids encoding *TXNIP* 3'UTR mutants (*TXNIP* 3'UTR MUT) and plasmid encoding the wild-type 3'UTR of *TXNIP* (*TXNIP* 3'UTR WT) as shown in Fig. 1E. miR-25-5p mimic negative control (mimic NC) and miR-25-5p mimic were co-transfected with *TXNIP* 3'UTR MUT or *TXNIP* 3'UTR WT, and TK vector was co-transfected at the same time, and the dual-luciferase reporter assay was performed 24 h later. The result indicated that compared with cells co-transfected with mimic NC and *TXNIP* 3'UTR WT, luciferase activity was significantly decreased in cells co-transfected with miR-25-5p mimic and *TXNIP* 3'UTR WT (Fig. 1F). In contrast, luciferase activity in cells co-transfected with miR-25-5p mimic with *TXNIP* 3'UTR MUT did not change significantly compared to cells co-transfected with mimic NC and *TXNIP* 3'UTR MUTs (Fig. 1F). The mimic NC, miR-25-5p mimic, miR-25-5p inhibitor and negative control of miR-25-5p inhibitor (inhibitor NC) were transfected into CTX TNA2 cells for 24 h, the relative RNA level of miR-25-5p was significantly overexpressed by the miR-25-5p mimic (*t* = 83.59, *P* < 0.0001), but the effect of miR-25-5p inhibitor on the relative RNA level of miR-25-5P was not significant (*t* = 0.6478, *P* = 0.5524, Fig. 1G). The relative mRNA level of *TXNIP* was significantly decreased by the miR-25-5p mimic (*t* = 33.26, *P* < 0.0001), and contrarily was significantly increased by miR-25-5p inhibitor (*t* = 15.29, *P* < 0.0001, Fig. 1H). When the CTX TNA2 cells were stimulated with LPS at 1 μg/mL, 2 μg/mL, and 5 μg/mL for 12 h, or with 2 μg/mL LPS for 0, 6, 12, and 24 h, the miR-25-5p RNA level was downregulated by 2 μg/mL and 5 μg/mL LPS (*F* = 8.06, *P* < 0.01), and by 2 μg/mL LPS at 6, 12, and 24 h (*F* = 78.96, *P* < 0.0001) (Fig. 1I,J), which is just the reverse of *TXNIP* (Fig. 1A,B). These results indicated that LPS upregulated the expression of TXNIP and downregulated the level of miR-25-5p, and that miR-25-5p negatively regulated the mRNA level of *TXNIP*.

### miR-25-5p alleviates reactive oxygen species (ROS) response, and cell apoptosis induced by LPS

**Figure 2:**
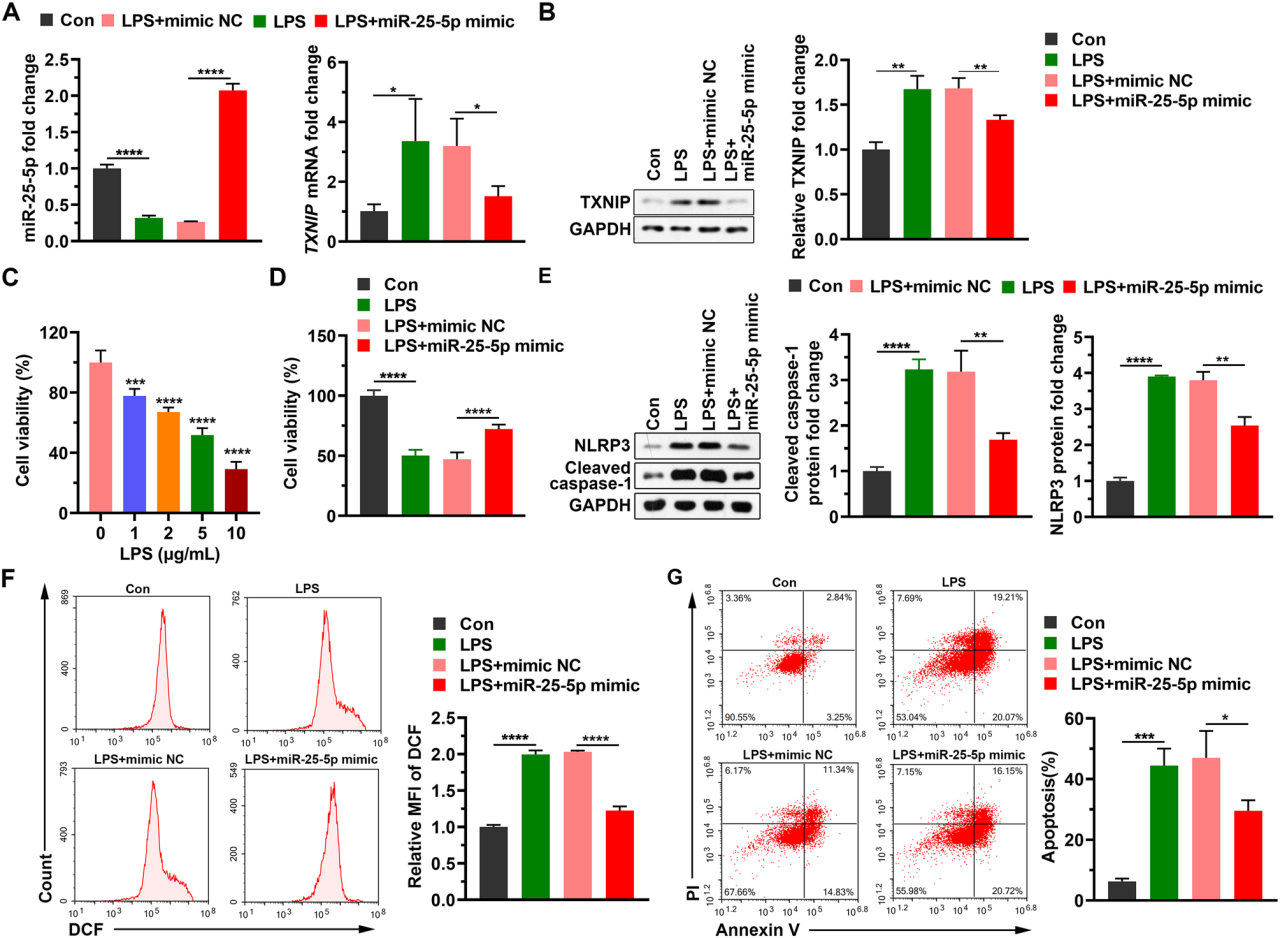
Neuroprotective effects of miR-25-5p on CTX TNA2 cells in response to LPS stimulation. (**A** and **B**) Effects of miR-25-5p mimic on miR-25-5p and *TXNIP* mRNA (A), TXNIP protein fold changes (**B**) of LPS-stimulated CTX TNA2 cells. (**C**) Cell viability of CTX TNA2 cells in response to 0 μg/mL, 1 μg/mL, 2 μg/mL, 5 μg/mL, and 10 μg/mL LPS stimulation at 12 h. (**D**–**G**) Effects of miR-25-5p mimic on cell viability (**D**), cleaved caspase-1 and NLRP3 protein fold changes determined by western blot (**E**), relative MFI of DCF (**F**), and percentage of apoptosis (**G**) of LPS-stimulated CTX TNA2 cells. The relative expression levels of miRNA and mRNA were determined by qPCR and protein levels were determined by Western blot. The independent *t*-test was applied to the comparison between groups (**A**, **B**, **D**–**G**). One-way analysis of variance (ANOVA), followed by Tukey’s test was used to identify the significant differences among multiple groups (**C**). Values are shown as the mean ± SD. Con: negative control of LPS, LPS: lipopolysaccharide, mimic NC: negative control of miR-25-5p mimic, PI: propidium iodide, MFI: mean fluorescence intensity. *P < 0.05. **P < 0.01. ***P < 0.001. ****P < 0.0001.

When CTX TNA2 cells transfected with mimic NC or miR-25-5p mimic for 24 h were treated with 2 μg/mL LPS for 12 h, the expression level of miR-25-5p significantly decreased by the addition of LPS (*t* = 19.15, *P* < 0.0001), which was significantly alleviated by miR-25-5p mimic (*t* = 34.69, *P* < 0.0001) (Fig. 2A), and the expression level of *TXNIP* mRNA and protein in the LPS group was significantly increased by the addition of LPS (*t* = 2.84, *P* < 0.05 for mRNA; *t* = 6.89, *P* < 0.01 for protein), which was significantly alleviated by miR-25-5p mimic (*t* = 2.97, *P* < 0.05 for mRNA; *t* = 4.84, *P* < 0.01 for protein) (Fig. 2A,B). When the CTX TNA2 cells were stimulated with LPS at 1 μg/mL, 2 μg/mL, and 5 μg/mL for 12 h, cell viability was significantly decreased by LPS at 1 μg/mL, 2 μg/mL, and 5 μg/mL (*F* = 154.2, *P* < 0.0001, Fig. 2C). CTX TNA2 cells transfected with mimic NC or miR-25-5p mimic for 24 h were treated with 2 μg/mL LPS for 12 h. The cell viability was significantly decreased by the addition of LPS (*t* = 18.45, *P* < 0.0001), which was significantly alleviated by miR-25-5p mimic (*t* = 8.97, *P* < 0.0001) (Fig. 2D). Similarly, the production of cleaved caspase-1 and NLRP3 in the LPS group was significantly stimulated by the addition of LPS (*t* = 16.31, *P* < 0.0001 for cleaved caspase-1; *t* = 51.25, *P* < 0.0001 for NLRP3), which was significantly decreased by miR-25-5p mimic (*t* = 5.36, *P* < 0.01 for cleaved caspase-1; *t* = 6.49, *P* < 0.01 for NLRP3) (Fig. 2E). The relative mean fluorescence intensity (MFI) of DCF was significantly stimulated by the addition of LPS (*t* = 28.20, *P* < 0.0001), which was significantly reduced by the miR-25-5p mimic (*t* = 23.72, *P* < 0.0001) (Fig. 2F). Similarly, the percentage of apoptosis was significantly increased by the addition of LPS (*t* = 11.56, *P* < 0.001), which was alleviated by the miR-25-5p mimic (*t* = 3.17, *P* < 0.05) (Fig. 2G). These data revealed that increasing the level of miR-25-5p alleviated LPS-induced decrease in cell viability, production of reactive oxygen species, and increase in apoptosis.

### miR-25-5p inhibits the secretion of inflammatory factors induced by LPS

**Figure 3:**
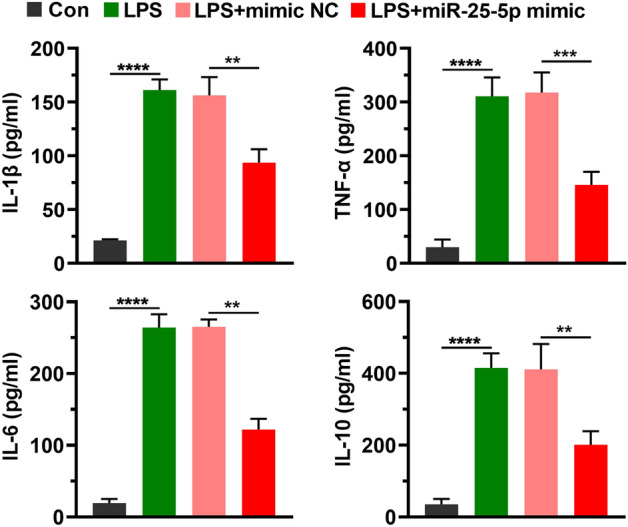
miR-25-5p alleviates inflammatory responses in LPS-stimulated CTX TNA2 cells. Effects of miR-25-5p mimic on IL-1β, TNF-α, IL-6, and IL-10 protein concentration determined by ELISA of LPS-stimulated CTX TNA2 cells. The independent *t*-test was applied to the comparison between groups. Values are shown as the mean ± SD. Con: negative control of LPS, LPS: lipopolysaccharide, mimic NC: negative control of miR-25-5p mimic. **P < 0.01. ***P < 0.001. ****P < 0.0001.

After CTX TNA2 cells transfected with mimic NC or miR-25-5p mimic for 24 h were treated with 2 μg/mL LPS for 12 h, the supernatant of the cultured cells was collected for enzyme-linked immunosorbent assay (ELISA). A significant increase was observed in the production of IL-1β (*t* = 28.16, *P* < 0.0001), IL-6 (*t* = 25.26, *P* < 0.0001), IL-10 (*t* = 17.56, *P* < 0.0001), and TNF-α (*t* = 14.90, *P* < 0.0001) by the addition of LPS, and the stimulated production was alleviated by the miR-25-5p mimic for IL-1β (*t* = 5.94, *P* < 0.001), IL-6 (*t* = 15.81, *P* < 0.0001), IL-10 (*t* = 5.257, *P* < 0.01), and TNF-α (*t* = 7.73, *P* < 0.001) (Fig. 3).

**Figure 4:**
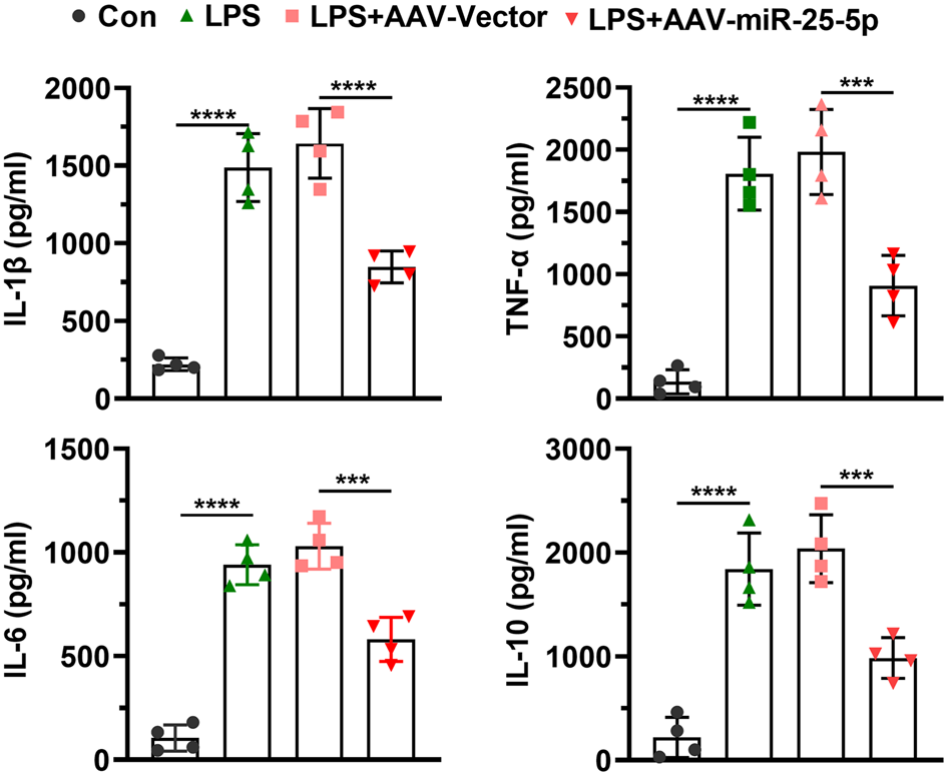
miR-25-5p alleviates inflammatory responses in LPS induced rats. Effects of AAV-miR-25-5p on serum concentrations of IL-1β, TNF-α, IL-6, and IL-10 determined by ELISA in septic rats. The independent *t*-test was applied to the comparison between groups. Values are shown as the mean ± SD. Con: negative control, LPS: lipopolysaccharide, AAV: adeno-associated virus. ***P < 0.001. ****P < 0.0001.

After rats were treated with LPS or/and AAV-miR-25-5p, the plasma was harvested for ELISA. A significant increase was observed in the concentration of IL-1β (*t* = 11.43, *P* < 0.0001), IL-6 (*t* = 14.47, *P* < 0.0001), IL-10 (*t* = 8.15, *P* < 0.001), and TNF-α (*t* = 10.83, *P* < 0.0001) after LPS injection, which was significantly alleviated by the injection of AAV-miR-25-5p for IL-1β (*t* = 6.46, *P* < 0.001), IL-6 (*t* = 5.87, *P* < 0.01), IL-10 (*t* = 5.53, *P* < 0.01), and TNF-α (*t* = 5.13, *P* < 0.001) (Fig. 4).

These findings implied that upregulation of miR-25-5p levels attenuated LPS-induced IL-1β, IL-6, IL-10, and TNF-α.

### miR-25-5p alleviates brain cell damage induced by LPS

**Figure 5:**
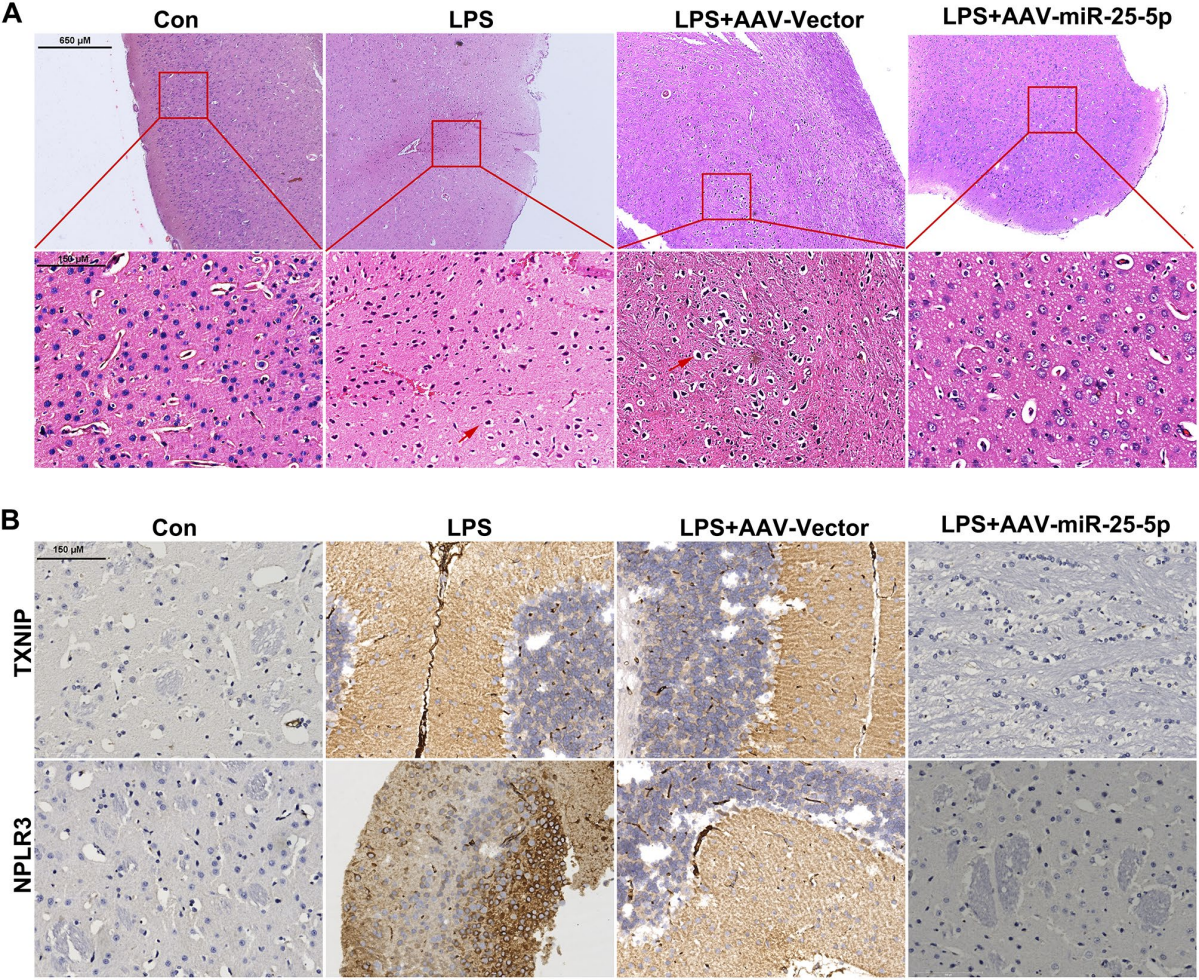
Neuroprotective effects of miR-25-5p on the brain of septic rats. (A and B) Effects of AAV-miR-25-5p on brain morphology of septic rats using HE (**A**), and TXNIP and NPLR3 signals on brain using IHC assay (**B**). Con: negative control, LPS: lipopolysaccharide, AAV: adeno-associated virus.

After rats were treated with LPS or/and AAV-miR-25-5p, the brain tissues were obtained for hematoxylin–eosin (HE) staining and immunohistochemical (IHC) assay. Normal morphology was observed in the brains of the control and LPS + AAV-miR-25-5p groups, and karyopyknosis was observed in the brains of the LPS and LPS + AAV-Vector group (Fig. 5A). The immunoreactivity of TXNIP and NLRP3 was detected in the brain of the LPS and LPS + AAV-Vector groups, but not in the brains of the control and LPS + AAV-miR-25-5p groups (Fig. 5B).

### miR-25-5p decreases the levels of TXNIP, catalase, MnSOD, NLRP3, and cleaved caspase-1 induced by LPS

**Figure 6:**
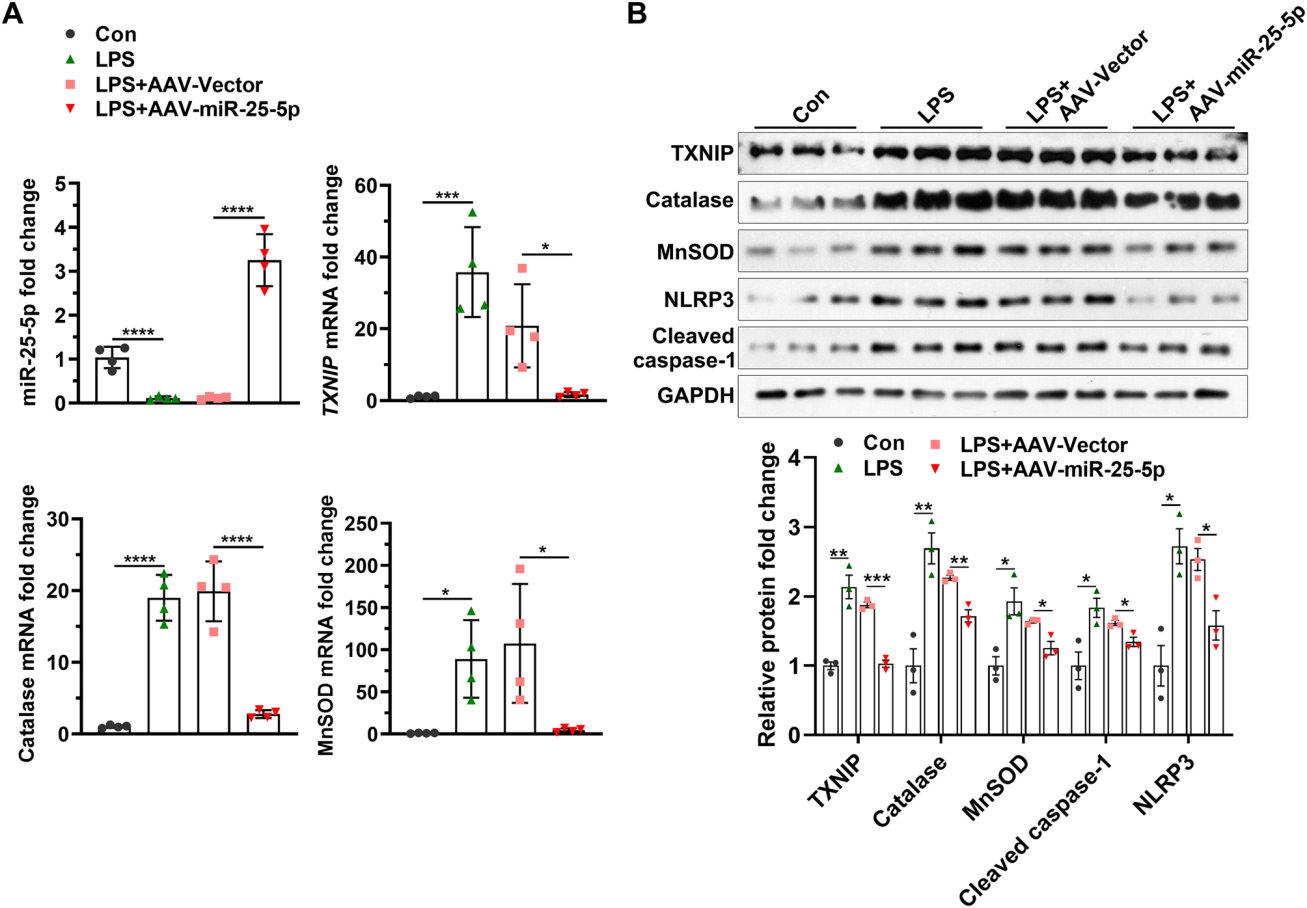
miR-25-5p negatively regulates signaling pathways in ROS response and apoptosis of the brain of septic rats. (**A**) Effects of AAV-miR-25-5p on miR-25-5p, *TXNIP*, *catalase*, and *MnSOD* mRNA fold changes (**A**), and TXINP, catalase, cleaved caspase-1, NLRP3, and MnSOD protein fold changes in the brain of septic rats (**B**). The relative expression levels of miRNA and mRNA were determined by qPCR and protein levels were determined by western blotting. The independent *t*-test was applied to the comparison between groups. Values are shown as the mean ± SD (**A**, and **B**). Con: negative control, LPS: lipopolysaccharide, AAV: adeno-associated virus. *P < 0.05. **P < 0.01. ***P < 0.001. ****P < 0.0001.

After rats were treated with LPS or/and AAV-miR-25-5p, the brain tissues were obtained for western blotting and qPCR. The miR-25-5p level in the brain of the control group was significantly higher than that in the LPS group (*t* = 7.48, *P* < 0.001), and miR-25-5p level in the brain of the LPS + AAV-miR-25-5p group was significantly greater than that in the LPS + AAV-Vector group (*t* = 10.60, *P* < 0.0001) (Fig. 6A). Similarly, significant stimulation of *TXNIP* (*t* = 5.54, *P* < 0.01), *catalase* (*t* = 11.18, *P* < 0.0001), and *MnSOD* (*t* = 3.83, *P* < 0.01) mRNA levels in the brain was observed in by the injection of LPS, while LPS-induced *TXNIP* (*t* = 3.31, *P* < 0.05), *catalase* (*t* = 8.13, *P* < 0.001), and *MnSOD* (*t* = 2.92, *P* < 0.05) mRNA levels were alleviated by the treatment of AAV-miR-25-5p (Fig. 6A). Consistently, the significant stimulation of the protein level in the brain was observed in TXINP (*t* = 6.35, *P* < 0.01), catalase (*t* = 5.14, *P* < 0.01), cleaved caspase-1 (*t* = 3.45, *P* < 0.05), NLRP3 (*t* = 4.46, *P* < 0.05), and MnSOD (*t* = 3.96, *P* < 0.05) by the injection of LPS, which was reduced by the treatment of AAV-miR-25-5p for TXINP (*t* = 12.99, *P* < 0.0001), catalase (*t* = 5.73, *P* < 0.01), cleaved caspase-1 (*t* = 3.60, *P* < 0.05), NLRP3 (*t* = 3.59, *P* < 0.05), and MnSOD (*t* = 3.92, *P* < 0.05) (Fig. 6B).

## Discussion

The *TXNIP* expression level was positively related to the concentration and time of LPS stimulation, which is in agreement with previous findings^20^. These findings confirmed the role of the TXNIP/NLRP3 pathway in the development of septic encephalopathy. From the miRDB, Targetscan, and microT-CDS databases, 10 miRNAs: miR-105, miR-106b-5p, miR-154-3p, miR-25-5p, miR-20b-5p, miR-295-3p, miR-291-3p, miR-301b, miR-352, and miR-93-5p, were predicted to target *TXNIP* mRNA. Among these miRNAs, the expression levels of miR-25-5p, miR-105, and miR-106b-5p were impacted by LPS stimulation. Especially for miR-25-5p, changes in the expression level were greatest and the expression profile was negatively related to the concentration and treatment time of LPS stimulation, in contrast to *TXNIP*. This evidence implied that miR-25-5p contributed to the regulation of the TXNIP/NLRP3 pathway. The regulative activity of miR-25-5p was confirmed for *TXNIP* expression by the luciferase reporter assay. The negative regulation of *TXNIP* expression by miR-25-5p indicated that the miR-25-5p mimic might be a candidate treatment for septic encephalopathy. Nonetheless, the regulative role of miR-105 and miR-106b-5p on *TXNIP* expression requires further investigation to verify.

TXNIP directly interacts with NLRP3 and is essential to activate the production of the NLRP3 inflammasome^23^. NLRP3 is a cytoplasmic signal receptor that activates caspase-1, which is involved in the maturation of IL-1β precursors^24^. Theoretically, the downregulation of the TXNIP/NLRP3 pathway by miR-25-5p can suppress inflammatory reactions. The hypothesis was verified by in vitro and in vivo experiments. Specifically, the IL-1β protein concentration was significantly reduced in LPS-stimulated CTX TNA2 cells by miR-25-5p mimic, and the IL-1β protein concentration was significantly decreased in the plasma of LPS-stimulated rats by injection of AAV-miR-25-5p. The reduction of the inflammatory cytokine level indicated that miR-25-5p alleviated inflammatory responses. Besides, Western blot analyses showed that TXNIP, NLRP3, and cleaved caspase-1 expression levels were significantly reduced in LPS-stimulated CTX TNA2 by the miR-25-5p mimic, and TXNIP, NLRP3, and cleaved caspase-1 expression levels in the brain were significantly alleviated by treatment of the AAV-miR-25-5p group. These findings confirmed that miR-25-5p downregulated inflammatory responses by the TXNIP/NLRP3 pathway. In contrast with IL-1β, IL-6, IL-8, and TNF-α are not downstream regulation targets of the TXNIP/NLRP3 pathway. Given that miRNAs typically regulate multiple genes^25^, the regulative target of miR-25-5p involving inflammatory responses merits further investigation.

The dysregulated inflammatory response can lead to tissue damage and organ dysfunction^3,4^. In agreement with previous findings, LPS stimulated a decrease of the cell viability and increase of the percentage of apoptosis in the CTX TNA2 cells, and abnormal morphology was observed in the brains of the LPS-stimulated group. Besides, the relative MFI of DCF was significantly increased by LPS stimulation in CTX TNA2 cells. The greater relative MFI of DCF indicated increased ROS production. Consistently, the increased protein level of MnSOD and catalase in the brains of the LPS group confirmed the ROS induced by LPS stimulation. The activation of oxidative stress likely causes the damage of cells and ROS production can be stimulated by inflammatory reactions^26^. Combined with the above findings, LPS activates the TXNIP/NLRP3 pathway and upregulates the inflammatory response, which leads to cell damage and tissue dysfunction.

Although we provided consistent results for the administration of miR-25-5p mimic or AAV-miR-25-5p 48 h after LPS stimulation. However, there is no substantial evidence to prove that LPS stimulation has the same effect on the central nervous system as sepsis, so this study cannot directly prove the effect of miR-25-5p on the central nervous system damage caused by sepsis, which is one of the limitations of this study. In addition, this study only investigated the role of miR-25-5p in the inflammation of CTX TNA2 cells, but failed to detect the role of miR-25-5p in the inflammation of other brain cells. Therefore, this study cannot perfectly clarify the mechanism of niR-25-5p in septicemia encephalopathy, which is also the limitation of this study.

In conclusion, in the present study, the cell viability was improved and relative MFI of DCF and percentage of apoptosis were decreased in CTX TNA2 cells by the miR-25-5p mimic treatment, and LPS-induced cell damage and abnormal morphology of the brain were alleviated by the treatment of AAV-miR-25-5p. The findings in the present study indicate that miR-25-5p plays a critical role in the inflammatory response of the brain, and miR-25-5p mimic or AAV-miR-25-5p have potential as therapeutic candidates for septic encephalopathy.

## Methods

### Animals

Twenty male Sprague-Dawley rats of clean-grade (200 g in weight) were purchased from Charles River (Beijing, China). The rats were housed in an animal dwelling that was kept at an ambient temperature of 23 °C and a humidity of 50%. The rats were exposed to 12 h of light per day. The animals were provided with unrestricted supplementation of food and water.

### Cell cultures

CTX TNA2 astrocyte cell line of the rats were purchased from IMMOCELL (Xiamen, Fujian, China). The cells were cultured in Dulbecco’s modified Eagle’s medium (Gibco, Detroit, MI, USA) with 100 U/mL streptomycin/penicillin, and 10% fetal bovine serum added 5% CO_2_ at 37 °C.

### Experimental design

To understand the anti-sepsis activity of miR-25-5p in *vitro*, CTX TNA2 cells were allocated randomly to four groups, which were treated with blank control (physiological saline), LPS, LPS + mimic negative control (mimic NC), and LPS + miR-25-5p mimic, respectively. After transfected 200 pmol miRNA mimic with transfection reagent for 24 h, 80% confluence of cells in 6-well plates were incubated with 2 μg/mL LPS for 12 h. To understand the anti-sepsis activity of miR-25-5p in *vivo*, the rats were randomly allocated to four groups after 14 days of acclimatization. After anaesthetization with 1% pentobarbital sodium, twenty were randomly divided into four groups, control group which were subjected to the injection (5 mg/kg) of blank control (physiological saline), LPS group, LPS + AAV-vector group, and LPS + AAV-miR-25-5p group, with 5 rats in each group. 5 mg/kg LPS and/or 1 × 10^12^ vg/kg of AAV-miR-25-5p were injected into the rat brain as previously described^27^.

### Ethics approval

All experiments were reported in accordance with ARRIVE guidelines^28^. All methods were carried out in accordance with relevant guidelines and regulations.

### miRNA target prediction

miRNAs potentially binding to *TXNIP* mRNA were searched against the miRNA database of miRDB (http://mirdb.org/), microT-CDS (http://diana.imis.athena-innovation.gr/DianaTools/index.php?r=microT_CDS/index), and Targetscan (http://www.targetscan.org/vert_71/), using the 3′-UTR sequence of *TXNIP*.

### Plasmid construction

The 3′-UTR sequence of *TXNIP* was amplified with the specific primers containing cleavage sites. After digestion with NotI and XhoI, the 3′-UTR sequence of *TXNIP* were recombined with the pmirGLO reporter vector (Promega, Madison, WI, USA). The QuikChange II Site-Directed Mutagenesis Kit (Agilent Technologies, CA, USA) was applied to synthesize mutant *TXNIP*-3ʹUTR plasmids. The primers are as follows. *TXNIP* 3′-UTR forward primer: 5′-GCTCGCTAGCCTCGAGGGCTCTTAAGGGTTAAGCCC-3′, *TXNIP* 3′-UTR reverse primer: 5′-ATGCCTGCAGGTCGACCGACACCTCCATCAGCTCAC-3′; *TXNIP* 3′-UTR mutant forward primer: 5′-AAACTTAAGAATAATTAGCACTTTGTTCCGTGTCC-3′, *TXNIP* 3′-UTR mutant reverse primer: 5′-TGCTAATTATTCTTAAGTTTTGGAGTGCTAGAGGC-3′.

### Luciferase reporter assay

mimic NC and miR-25-5p mimic were co-transfected with *TXNIP* 3'UTR MUT or *TXNIP* 3'UTR WT using Lipofectamine 2000 reagent into CTX TNA2 cells, and TK vector was co-transfected at the same time. After lysis at 24 h post-transfection, the luciferase activities (firefly luciferase activity and Renilla luciferase activity) were determined by the Dual Luciferase Reporter Assay System (Promega, USA) and the former was normalized to the latter to estimate the expression level.

### Assay of cell proliferation

The treated CTX TNA2 cells were seeded into 96-well plates. The density per well was approximately 10^4^ cells. After the incubation with a cell counting kit 8 solution, the viability of the CTX TNA2 cells was determined by an absorbance reader (SpectraMax, San Francisco, CA, USA) at an absorbance of 450 nm.

### Assay of ROS

To detect the ROS, the treated CTX TNA2 cells were stained with 10 μmol/L DCFH-DA (catalog number: S0033S, Beyotime). The cells were then diluted with RPMI 1640 medium. The ROS intensity was measured by a flow cytometer (ACEA, San Diego, CA, USA).

### Assay of cell apoptosis

The treated CTX TNA2 cells were incubated with annexin V-fluorescein isothiocyanate and propidium iodide reagents (Vazyme, Nanjing, China) following the manufacturer’s instruction. The apoptotic cells were determined by a flow cytometer (ACEA, San Diego, CA, USA) and the proportion of apoptotic cells was calculated.

### Sampling of blood and brain

After 48 h post-injection, blood was sampled from the tail vein of the rats. The blood sample was centrifuged for 10 min at 3000×*g* to obtain plasma. The rats were then euthanized with pentobarbital and the brain was immediately sampled after dissection. After collection, the blood and brain samples were immediately frozen in liquid nitrogen. The frozen samples were transferred to a − 80 °C freezer for storage until further analyses.

### HE staining

For histological observation, the brain sample was fixed with 10% formalin. The washed sample was embedded in paraffin, followed by sectioning (5 μm). The sections were stained by HE to obtain HE slides. The slides were observed by an Olympus light microscope (BX51, Olympus corporation).

### IHC assay

For immunohistochemical observation, the sections of the brain sample were deparaffinized, rehydrated, and treated with 3% H_2_O_2_, followed by the addition of citrate buffer. The blocked sections were incubated with TXNIP antibody (catalog number: 18243-1-AP, Proteintech, Wuhan, China), or NLRP3 antibody (catalog number: 19771-1-AP, Proteintech), followed by incubation with the HRP-conjugated goat anti-rabbit IgG (catalog number: SA00001-2, Proteintech). The sections were subsequently treated with a 3,3-diaminobenzidine substrate system. The slides were observed using an Olympus light microscope (BX51, Olympus corporation).

### An enzyme-linked immunosorbent assay (ELISA)

Rat IL-1β/IL-1F2 ELISA Kit (catalog number: RLB00, China Bio-Techne China Co., Ltd.), Rat IL-6 ELISA Kit (catalog number: R6000B, China Bio-Techne China Co., Ltd.), Rat IL-10 ELISA Kit (catalog number: R1000, China Bio-Techne China Co., Ltd.), Rat TNF-α ELISA Kit (catalog number: RTA00, China Bio-Techne China Co., Ltd., Shanghai, China) were applied to determine the protein concentration of IL-1β, IL-6, IL-10, and TNF-α, and the protein concentration was determined by a microplate reader (BMG Labtech, VIC, Australia) at an absorbance of 450 nm, following the manufacturer’s instructions. The variations of intra- and inter-assay were both < 10% in the present study.

### Western blotting

The proteins of the brain samples were isolated with RIPA lysis buffer, which is composed of protease, phosphatase inhibitors, and PMSF (Beyotime, Shanghai, China). After separation by SDS-PAGE gel, the proteins were transferred to polyvinylidene difluoride membranes (Bio-Rad, Hercules, CA, USA), which was blocked with 5% non-fat milk. The membrane was incubated with primary antibodies (anti-TXNIP, anti-catalase, anti-MnSOD, anti-caspase-1, and anti-NLRP3) and HRP-conjugated secondary antibodies. The HRP chemiluminescence kit (Immun-StarTM, Bio-Rad) was used to visualize protein bands on the ImageQuant LAS 4000 system (GE Healthcare, Hino, Japan). Primary antibodies were TXNIP antibody (catalog number: 18243-1-AP, Proteintech, Wuhan, China), catalase antibody (catalog number: 21260-1-AP, Proteintech), MnSOD antibody (catalog number: ab13533, Abcam, Shanghai, China), caspase-1 antibody (catalog number: #4199, Cell Signaling Technology, Shanghai, China), NLRP3 antibody (catalog number: ab214185, abcam), GAPDH (catalog number: 21260-1-AP, Proteintech). Secondary antibodies were HRP-conjugated Goat Anti-mouse IgG(H + L) (catalog number: SA00001-1, Proteintech) and HRP-conjugated Goat Anti-Rabbit IgG(H + L) (catalog number: SA00001-2, Proteintech). GAPDH was chosen as an internal control for western blotting.

### RNA extraction and first-strand cDNA synthesis

The total RNA was extracted using TRIzol reagents (TaKaRa) for mRNAs and the miRVanaTM miRNA isolation kit (Ambion, Austin) for miRNAs, following manufacturer’s instructions. The quality of extraction was assessed by agarose gel electrophoresis and UV spectrophotometry. The total RNA was treated with DNase I (TaKaRa). The mRNAs and miRNAs were reverse-transcribed using commercial kits (TaKaRa), according to manufacturer’s instructions.

### Real-time quantitative PCR

Real-time quantitative PCR (qPCR) was applied to determine the expression level of mRNA and miRNA in a QuantStudio 3 Real-time PCR System (Thermo Scientific, MA, USA) using TB Green Premix Ex TaqTM II (TaKaRa, Dalian, China). The PCR reaction was programmed as per the following parameters: preheating at 95 °C for 60 s, 40 repeated cycles of heating at 95 °C for 30 s, cooling at 58 °C for 35 s, extension at 72 °C for 60 s, and a final extension at 72 °C for 10 min. The relative expression level was calculated using the 2^−ΔΔCT^ algorithm (Livak and Schmittgen 2001), in which glyceraldehyde-3-phosphate dehydrogenase was used as the internal control. 18 s RNA was selected as an internal control for qPCR of *TXNIP*, Catalase, and MnSOD. U6 was chosen as an internal control for qPCR of miRNAs. Information on the PCR primers is provided in the “Supplementary material”. rno-miR-291a-3p reverse transcription primer: 5′-GTCGTATCCAGTGCAGGGTCCGAGGTATTCGCACTGGATACGACGGCACA-3′, rno-miR-291a-3p forward primer: 5′-GCGAAAGTGCTTCCACTTTG-3′; rno-miR-295-3p reverse transcription primer: 5′-GTCGTATCCAGTGCAGGGTCCGAGGTATTCGCACTGGATACGACACACCC-3′, rno-miR-295-3p forward primer: 5′-CGCGCGAAGTGCTACTACTTTT-3′; rno-miR-106b-5p reverse transcription primer: 5′-GTCGTATCCAGTGCAGGGTCCGAGGTATTCGCACTGGATACGACATCTGC-3′, rno-miR-106b-5p forward primer: 5′-GCGCGTAAAGTGCTGACAGT-3′; rno-miR-154-3p reverse transcription primer: 5′-GTCGTATCCAGTGCAGGGTCCGAGGTATTCGCACTGGATACGACAATAGG-3′, rno-miR-154-3p forward primer: 5′-CGCGAATCATACACGGTTGA-3′; rno-miR-25-5p reverse transcription primer: 5′-GTCGTATCCAGTGCAGGGTCCGAGGTATTCGCACTGGATACGACGCAATT-3′, rno-miR-25-5p forward primer: 5′-AGGCGGAGACACGGGC-3′; rno-miR-301b-5p reverse transcription primer: 5′-GTCGTATCCAGTGCAGGGTCCGAGGTATTCGCACTGGATACGACAGTAGT-3′, rno-miR-301b-5p forward primer: 5′-GCGGCTCTGACTAGGTTGC-3′; rno-miR-20b-5p reverse transcription primer: 5′-GTCGTATCCAGTGCAGGGTCCGAGGTATTCGCACTGGATACGACCTACCT-3′, rno-miR-20b-5p forward primer: 5′-GCGCAAAGTGCTCATAGTGC-3′; rno-miR-352 reverse transcription primer: 5′-GTCGTATCCAGTGCAGGGTCCGAGGTATTCGCACTGGATACGACTACTAT-3′, rno-miR-352 forward primer: 5′-CGCGCGAGAGTAGTAGGTTGC-3′; rno-miR-105 reverse transcription primer: 5′-GTCGTATCCAGTGCAGGGTCCGAGGTATTCGCACTGGATACGACACCACA-3′, rno-miR-105 forward primer: 5′-CGCGCAAGTGCTCAGATGTC-3′; rno-miR-93-5p reverse transcription primer: 5′-GTCGTATCCAGTGCAGGGTCCGAGGTATTCGCACTGGATACGACCTACCT-3′, rno-miR-93-5p forward primer: 5′-CGCAAAGTGCTGTTCGTGC-3′; common reverse primer: 5′- AGTGCAGGGTCCGAGGTATT-3′; rat *TXNIP* forward primer: 5′-CAATACTCCTGACTTAATGG-3′, rat *TXNIP* reverse primer: 5′-TCATCACCTTCACAGAAT-3′; rat *MnSOD* forward primer: 5′-GCCATATCAATCACAGCATT-3′, rat *MnSOD* reverse primer: 5′-CCAGCAACTCTCCTTTGG-3′; rat *Catalase* forward primer: 5′-GAATGGCTATGGCTCACA-3′, rat *Catalase* reverse primer: 5′-TGGTCAGTCTTGTAATGGAA-3′; 18 s RNA forward primer: 5′-AGGCGCGCAAATTACCCAATCC-3′, 18 s RNA reverse primer: 5′-GCCCTCCAATTGTTCCTCGTTAAG-3′; U6 reverse transcription primer: 5′-AACGCTTCACGAATTTGCGT-3′, U6 forward primer: 5′-CTCGCTTCGGCAGCACA-3′, U6 reverse primer: 5′-AACGCTTCACGAATTTGCGT-3′.

### Statistical analysis

All statistical analyses were conducted on Graph Pad Prism 3.0 software (San Diego, California, USA). The independent *t*-test was applied to the comparison between groups, and the normality of data was verified by the Shapiro–Wilk test. One-way analysis of variance (ANOVA), followed by Tukey’s test was used to identify the significant differences among multiple groups. A significant difference was considered when *P* < 0.05. All cell experiments were performed independently for more than three times.

## Supplementary Information

Supplementary Information.

## Data Availability

The data of miRNAs are available from miRDB (http://mirdb.org/), microT-CDS (https://dianalab.e-ce.uth.gr/html/dianauniverse/index.php?r=microT_CDS), and Targetscan (https://www.targetscan.org/vert_72/). Other materials used during the present study are available from the corresponding author on reasonable request.
