# Supplementary figures and images for: MicroRNA-25-5p negatively regulates TXNIP expression and relieves inflammatory responses of brain induced by lipopolysaccharide
Source: Sci Rep. 2022 Oct 26;12:17915. doi: 10.1038/s41598-022-21169-5 (PMC9605969; doi:10.1038/s41598-022-21169-5)

Figure 2B

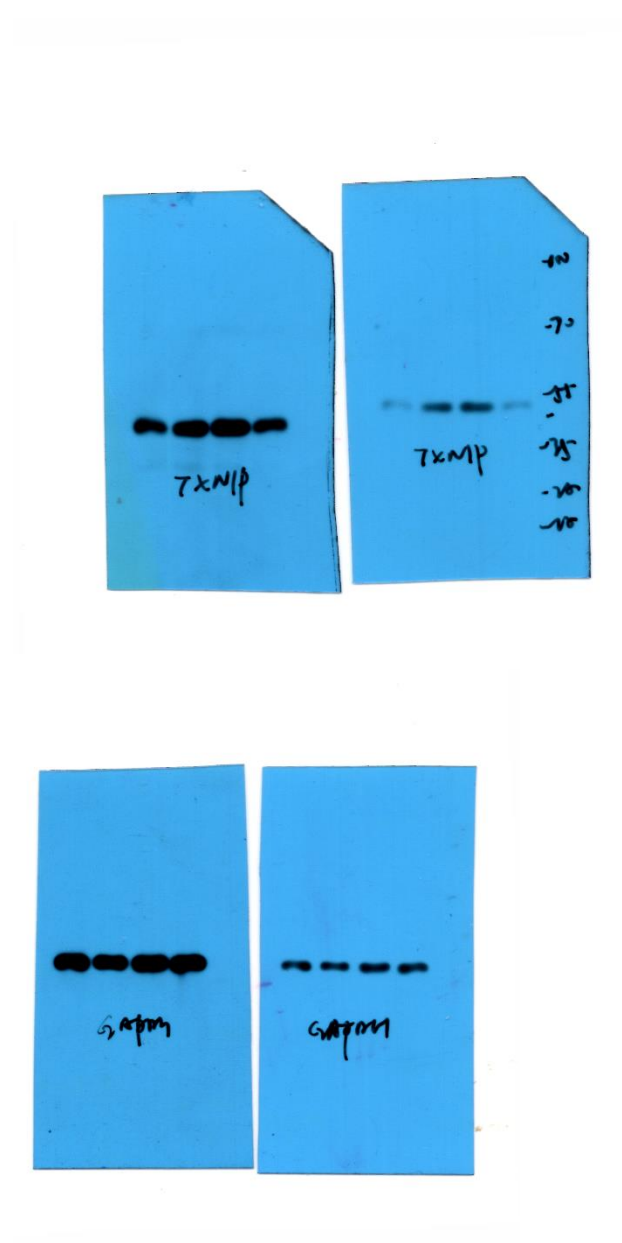

Figure 2E

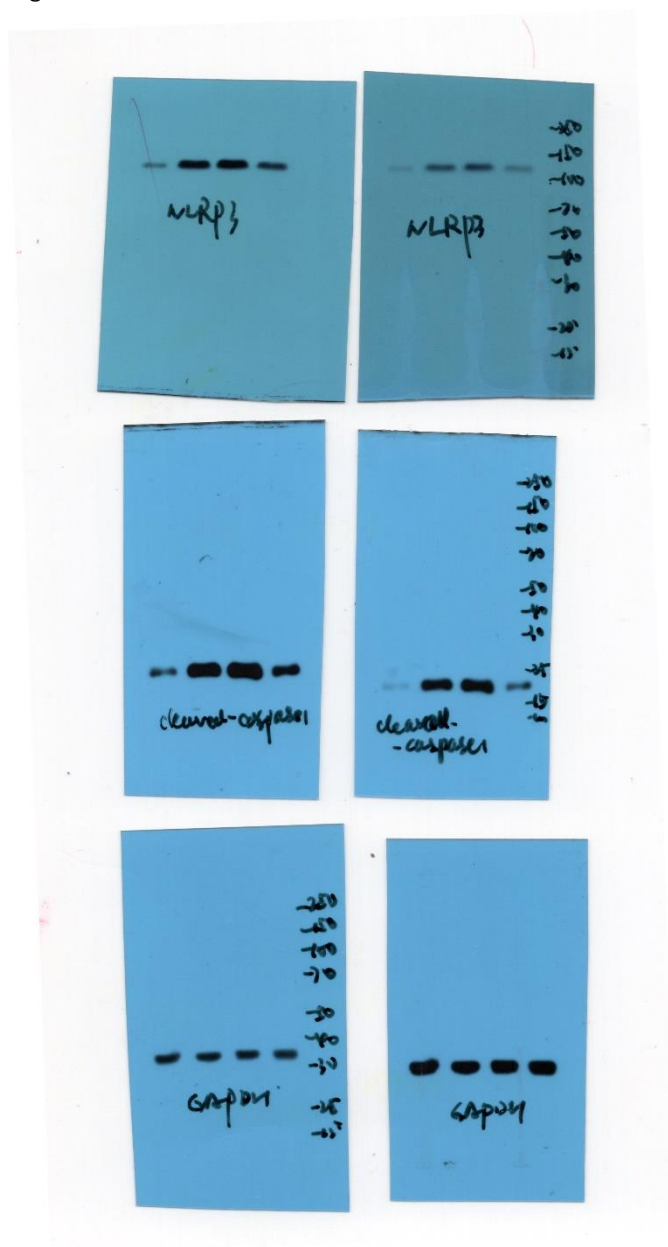

Figure 6B

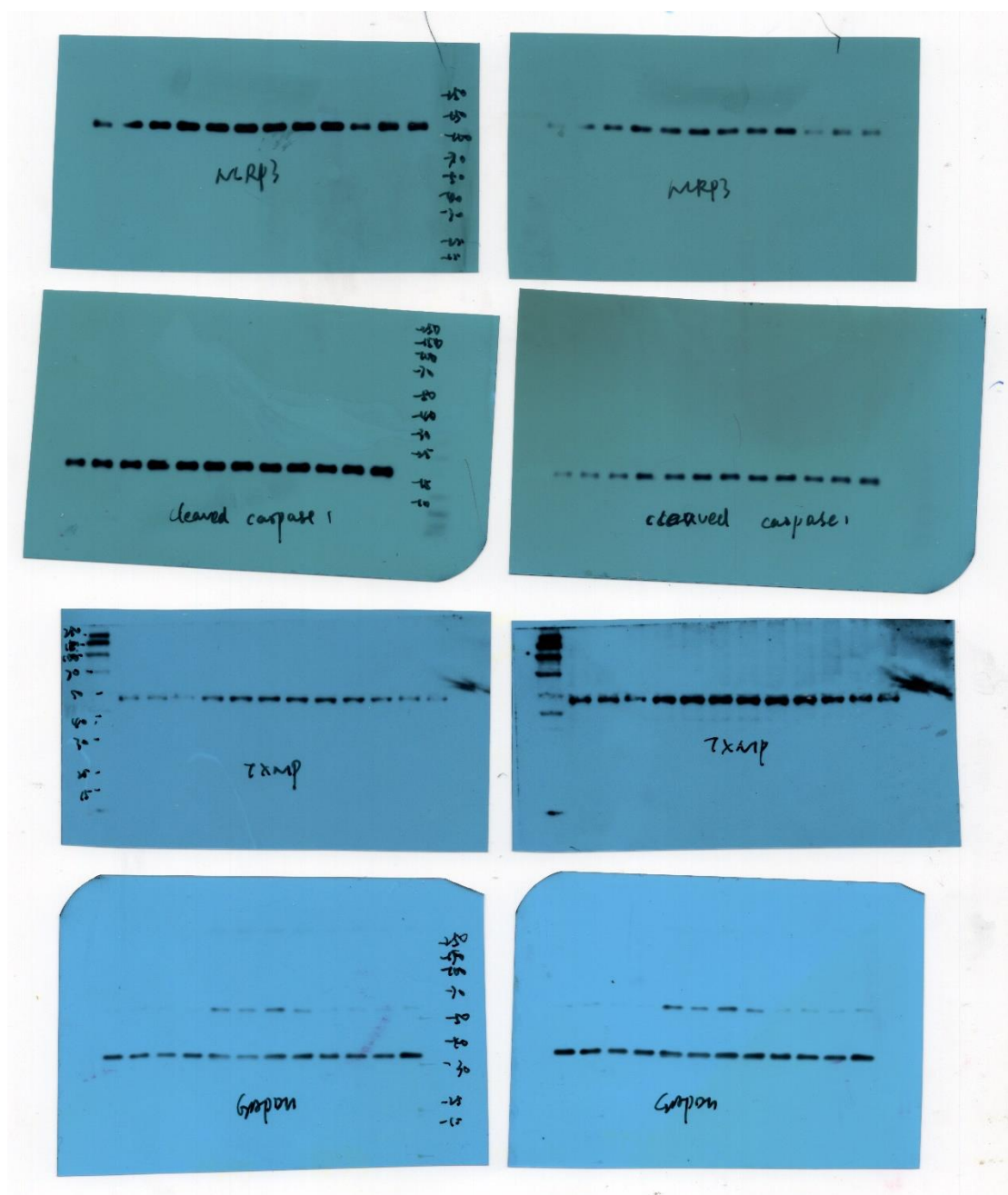

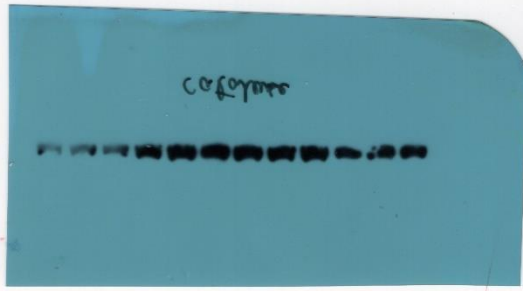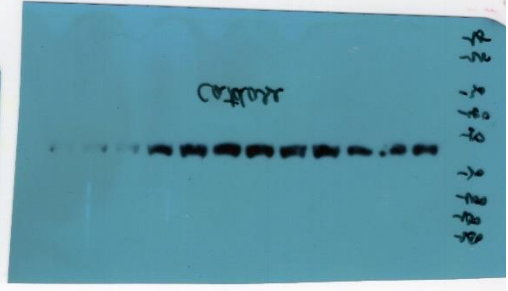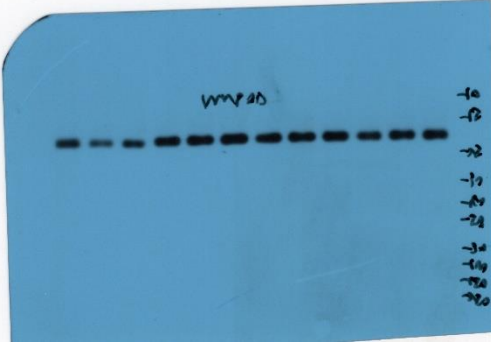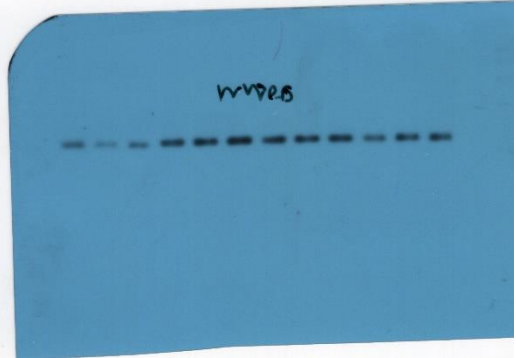

Supplement: Supplementary file 1 — Supplementary Information. [file 41598_2022_21169_MOESM1_ESM.pdf]
